# Correction: Prevalence and factors influencing drug-resistant tuberculosis in four regions of Ghana

**DOI:** 10.1371/journal.pone.0343420

**Published:** 2026-02-19

**Authors:** Esther Ba-Iredire, James Atampiiga Avoka, Luke Abanga, Abigail Awaitey Darkie, Emmanuel Junior Attombo, Eric Agboli

There is an error in affiliation 6 for authors Eric Agboli. The correct affiliation 6 is: Bernhard Nocht Institute for Tropical Medicine, Hamburg, Germany.
