# Supplementary material for: Prevalence and factors influencing drug-resistant tuberculosis in four regions of Ghana
Source: PLoS One. 2025 Sep 9;20(9):e0331958. doi: 10.1371/journal.pone.0331958 (PMC12419598; doi:10.1371/journal.pone.0331958)
Supplement: Attachment — Submitted filename: Response to reviewers.docx [file pone.0331958.s003.docx]

Lower Manya Krobo Municipal Health Directorate

P.O.Box 46

Odumase Krobo-Ghana

31/10/2024

The Editor

PLOS ONE Journal

Dear Sir/Madam,

**SUBMISSION OF REVISED MANUSCRIPT TITLED**: **PREVALENCE AND FACTORS INFLUENCING DRUG-RESISTANT TUBERCULOSIS IN FOUR REGIONS OF GHANA, PONE-D-24-05094**

This is to submit to you the responses and revised version of the manuscript number **PONE-D-24-05094, titled: Prevalence and factors influencing drug-resistant tuberculosis in four regions of Ghana.** One manuscript has the tracked version of the corrections and the other is unmarked.

Kindly take note that the data have been re-analyzed and updated to suit the standards of this journal.

We hope to hear favorably from you soon.

*Signed*

**James Atampiiga Avoka (PhD)**

**Corresponding author**

**Methods**

- Clearly identify the study population for determination of prevalence

**Response**

- The study population used for the determination of prevalence was the total number of confirmed TB cases (high-risk group) in the four study sites. Therefore, the prevalence in this case was calculated as all DR-TB cases identified divided by the total number of high-risk populations in the study areas (Confirmed TB cases).
- clearly state the model of analysis that help to determine cOR, aOR, p-values

**Response**

A logistic regression model was used to determine the crude and adjusted odds ratios and their corresponding p-values at 5% confidence interval.

- As stated in the Manuscript - the main outcome variable was the prevalence of DR-TB in the four study sites (what is the other outcome variable (if any)? and What was the possible categories in prevalence determination?)

**Response**

The main outcome variable in the study was prevalence of DR-TB. The other objective sought to determine the association between underweight and adverse drug reaction and treatment outcome. Therefore, the other outcome variable was prevalence of underweight DR-TB cases. The prevalence of underweight DR-TB in the four study sites is 6.73%.

- As stated in the Manuscript - all susceptible TB cases were also extracted but as cases of interest. (what was the denominator to calculate the prevalence?)

**Response**

The denominator was all confirmed TB cases in each study area.

**Result**

- - Title of table1a and 1b not clear (reported TB cases (1038) vs TB cases (839) should be clearly define). The two table can be merged and should have complete title description

**Response**

Title on tables 1a and 1b have been made clearer and merged.

- Table 2,3 & 4 have the same objective - better to re analyze all independent variables with outcome variable and produce one table containing cOR, aOR and p-value rather than three tables

**Response**

All independent variables with outcome variables re-analyzed and combined into one Table.

**Discussion**

- remove subtitles under discussion section

**Response**

All subtitles under the discussion section removed.

**Reference**

- the references were not exhaustive e.g. Sambas etal was missed

**Response**

References updated to include Sambas et al.

Top of Form

Reviewer 1

Summary
1. The second sentence should be excluded. Not needed.

**Response**

Second sentence excluded

2. It is not explained what the abbreviation BMI, SHS means.

**Response**

Abbreviations explained. BMI-Body Mass Index, SHS-Senior High School

3. Is there a significant correlation between minor adverse drug reaction and major adverse drug reaction variable?

**Response**

There is a significant correlation between DR-TB and major and minor adverse drug reactions. For major adverse drug reaction, (aOR 125.503, p-value <0.000), minor adverse drug reaction (aOR 23.597, p-value<0.000).

4. When writing the p value, please enter ˂0.0001 in all places in the introduction.

**Response**

P-value corrected.

5. There is an association between BMI and adverse reactions, please indicate whether lower or higher BMI value. The clear connection described is not enough. It is seen only through the conclusion.

**Response**

For the BMI, we meant underweight-It`s been corrected

6. Enter commas between the minimum and maximum values of the confidence interval.

**Response**

Corrections effected

Methods
Well described. It is necessary to describe and explain BMI in the "data extraction" part, and not to be described in the "data analysis" part.

**Response**

BMI explained in the data extraction part.

Transfer data about BMI to "data extraction". Define which are dependent and which are independent variables within regression analysis. There are no clearly defined categories for all the variables included in the research, and for outcomes. Also, not all outcomes are explained, e.g. major and minor adverse reactions…….

**Response**

All variables explained under study variables.

Define the method of calculating the prevalence….

**Response**

The prevalence was calculated as all DR-TB cases identified divided by the total number of high-risk populations in the study areas (Confirmed TB cases).

Prevalence=	**DR-TB cases**

**Diagnosed TB cases**

Results

It is necessary to express all percentages in the text and tables with numbers with one decimal place. Please correct. Attachment titles are not adequate.
Join table one and b. Correct the title of the table 1 and b.

For example - Demographic characteristics, habits and other characteristics of persons with TB in 4 regions of Ghana.

Table 2 and 3 have the same title. You must enter a number and percentage for each variable in Tables 2, 3, and 4.

No data is entered on graph 1 regarding the x and y axes.
Under each table enter explanations for abbreviations, statistical tests

**Response**

All issues under results have been resolved.

Discussion

According to the research results. It would be very important if you explained the data analysis in the "Methods" section, just as you divided the discussion of the results into three parts.

**Response**

Data analysis were explained under the data analysis part.

Please remove the first two sentences from the discussion.

**Response**

First two sentences removed.

Literature
Number of references greater than 30. Adequately cited.
Correct everywhere the way of citing multiple references in the work. For example (5-9) or (5,7,11).

**Response**

All references corrected.

**Reviewer #2:**

1) Retrospective analysis of DR-TB data from four regions of Ghana were carried out. To determine prevalence, defining the population base in which the outcome is determined is important - sampling method. Authors need to clearly state how the DR-TB cases were sampled. As written, I had to assume that DR-TB cases were measured among all TB cases. If this is the case, it needs to be stated clearly. presenting at various treatment centers in the four regions.

**Response**

The issue of prevalence calculation and denominator has been dealt with in the revised version. The study was a retrospective review of DR-TB cases from 2018 to 2022.

2) How are the four regions selected. How many regions are there? How many treatment centers were included? Answering these questions are important from generalizability perspective. If a convenience sampling approach was taken, it needs to be stated.

**Response**

The study sites were divided into four zones: The Northern, Southern, Coastal, and Middle Belts. The Bono, Western, Ashanti, Brong Ahafo, and Ahafo regions made up the middle belt. The Western and Central Regions were part of the coastal belt. The Northern, Savanna, Upper East, and North East Regions were all part of the Northern Belt. The Eastern, Volta, Oti, and Greater Accra regions made up the southern belt. Following that, a random selection of treatment centers was made from each of the four clusters to include them in the study. Additionally, in each of the selected regions, the central treatment centers were purposefully selected for the study. This is because all the DR-TB cases report to those treatment centers for management.

3) The inclusion criteria states that all DR-TB cases in four regions of Ghana. It also states that all susceptible TB cases were also extracted as cases of interest. The phrasing should be revised appropriately. One way to revise may be, 'all DR-TB cases out of all TB cases registered......". If including the susceptible new cases are optional, then how is the prevalence of DR-TB determined? What is the denominator? The exclusion criteria states, 'records of DR-TB cases from 2017 and below and 2023 were excluded'. Improve the english by using the words before 2017 or after 2023 instead of 'below'.
**Response**

Corrections effected.

4) If determining risk factors for development of DR-TB is an objective, then including only DR-TB patients cannot allow the determination, as there is no comparator. How the statistical analysis is carried out to determine risk factors is not described. If the authors are meaning to say that sociodemographic and clinical risk factors associated with the development of DR-TB among all TB cases was assessed, then it should be stated clearly that the risk factors were compared between people that did and not have DR-TB.

**Response**

Adequate description on how the analysis was conducted to determine risk factors has now been added. The study included both DR-TB and normal TB cases.

5) No mention was made of Primary drug resistant TB. Do the DR-TB cases including both Primary and acquired DR-TB cases? If Primary DR-TB is included, then how are the association of adverse drug reactions with development of DR-TB relevant, since these primary DR-TB patients have not received any drug at the time of diagnosis.

**Response**

This study included only DR-TB cases that were already on treatment. This shows why the study extracted data generated from cohorts 2018 to 2022.Those that were newly diagnosed and not on treatment were not part of this study.

6) It appears that diagnosis was happening through Xpert only. Are there any validation by culture or LPA? If diagnosis is only Xpert, RR-TB would be appropriate term.

**Response**

Results:
1) Overall, the results would need to be better presented.

**Response**

The results have been re-analyzed and presented afresh.

2) Prevalence of DR-TB was described for 3 regions, Ashanti, Eastern and Central. What is the fourth region?

**Response**

The fourth region, Upper West region has now been described.

3) Prevalence of DR-TB should be stratified by Primary Drug resistance or at least DR-TB among new cases and then DR-TB among retreatment cases.

**Response**

Unfortunately, the data were not disaggregated into new and retreated DR-TB cases. Hence, it would not be possible to determine that at this time.

4) What is SHS level of education? Why are more educated people more likely to have DR-TB

**Response**

SHS is Senior High School. This group is shown to be more likely to have DR-TB probably due to their youthful exuberance and risky behaviors. Also, the youth are usually carefree and do not adhere to instructions especially during treatment of TB.

Discussion:
I am surprised that the discussion has no mention of the impact of Covid-19 on the case detection of DR-TB.

**Response**

Discussion on impact of Covid-19 on case detection has now been included.

6) It appears that diagnosis was happening through Xpert only. Are there any validation by culture or LPA? If diagnosis is only Xpert, RR-TB would be appropriate term.

**Response**

Currently, DR-TB cases in Ghana are detected using the GeneExpert machine as first line and culture and sensitivity test (DST) for the confirmatory test.
