# Supplementary material for: Prevalence and factors influencing drug-resistant tuberculosis in four regions of Ghana
Source: PLoS One. 2025 Sep 9;20(9):e0331958. doi: 10.1371/journal.pone.0331958 (PMC12419598; doi:10.1371/journal.pone.0331958)
Supplement: Attachment — Submitted filename: Response_to_reviewers_auresp_2.docx [file pone.0331958.s004.docx]

Lower Manya Krobo Municipal Health Directorate

P.O.Box 46

Odumase Krobo-Ghana

02/07/2025

The Editor

PLOS ONE Journal

Dear Sir/Madam,

**SUBMISSION OF REVISED MANUSCRIPT TITLED**: **PREVALENCE AND FACTORS INFLUENCING DRUG-RESISTANT TUBERCULOSIS IN FOUR REGIONS OF GHANA, PONE-D-24-05094R1**

This is to submit to you the responses and revised version of the manuscript number **PONE-D-24-05094R1, titled: Prevalence and factors influencing drug-resistant tuberculosis in four regions of Ghana.** One manuscript has the tracked version of the corrections and the other is unmarked.

We hope to hear favorably from you soon.

*Signed*

**James Atampiiga Avoka (PhD)**

**Corresponding author**

**PONE-D-24-05094R1**

Prevalence and Factors Influencing Drug-Resistant Tuberculosis in four Regions of Ghana

PLOS ONE

• The manuscript indicates that GeneXpert was the primary method used to validate DR-TB. However, the text does not specify what proportion of the study population underwent this testing. What percentage of the total population was tested for susceptibility to anti-TB drugs?

**Response**

Since the percentage of the population screened for anti-TB medication susceptibility was not one of our variables of interest, it was not calculated. The prevalence of DR-TB and its risk factors were the only things that the writers were interested in.

• Based on the text and the conclusions drawn, I strongly assume that only a small fraction of TB patients underwent these tests. Therefore, the prevalence of DR-TB should be calculated using only those cases that were tested.

**Response**

The data from the DR-TB treatment centres was used to determine the prevalence of DR-TB. For all presumed TB cases, including DR-TB, the GeneXpert test is currently the primary first-line test in Ghana.

• It is inappropriate to discuss alcohol consumption as a risk factor without further clarification, as alcohol consumption can vary widely in terms of quantity. It should be categorized by the amount consumed.

**Response**

The authors of this retrospective study only retrieved data that was available at the time of the investigation. The alcohol intake amounts were not included in the records. Most likely, we'll record it as a limitation.

• Marital status or school attendance, considered indicators of close contact status, do not in themselves constitute risk factors for the onset or development of DR-TB. Close contact with an infected individual is a significant risk factor for exposure to the TB bacteria.

**Response**

Correction: The main risk factors for exposure to tuberculosis are contact with infected partners or classmates, not marital status or attendance at school.

• The term "chronic condition" is not well-defined in the manuscript. Chronic diseases can differ greatly and have varying effects on the immune system, either directly or through the medications prescribed for them. They should not be grouped to that further specificity.

**Response**

Chronic condition in this study was defined as hypertension, diabetes, HIV and cardiovascular diseases.

• It is unclear what is meant by "male class" and "female class." Is being classified as "male" or "female" itself a risk factor for DR-TB in the absence of other associated risk factors?

**Response**

One of the main risk factors for DR-TB is being male or female. Males are more vulnerable not only because they are men but also because of their exposure to dangerous behaviours like drinking, smoking, working in hazardous locations, and engaging in poor health-seeking behaviours. In contrast to males, females are more cautious and don't engage in risky behaviours.
