# Supplementary material for: Prevalence and factors influencing drug-resistant tuberculosis in four regions of Ghana
Source: PLoS One. 2025 Sep 9;20(9):e0331958. doi: 10.1371/journal.pone.0331958 (PMC12419598; doi:10.1371/journal.pone.0331958)
Supplement: Attachment — Submitted filename: Response to reviewers 3.docx [file pone.0331958.s005.docx]

Lower Manya Krobo Municipal Health Directorate

P.O.Box 46

Odumase Krobo-Ghana

21/08/2025

The Editor

PLOS ONE Journal

Dear Sir/Madam,

**SUBMISSION OF REVISED MANUSCRIPT TITLED**: **PREVALENCE AND FACTORS INFLUENCING DRUG-RESISTANT TUBERCULOSIS IN FOUR REGIONS OF GHANA, PONE-D-24-05094R2**

This is to submit to you the responses and revised version of the manuscript number **PONE-D-24-05094R2, titled: Prevalence and factors influencing drug-resistant tuberculosis in four regions of Ghana.** One manuscript has the tracked version of the corrections and the other is unmarked.

We hope to hear favorably from you soon.

*Signed*

**James Atampiiga Avoka (PhD)**

**Corresponding author**

**PONE-D-24-05094R2**

Prevalence and Factors Influencing Drug-Resistant Tuberculosis in four Regions of Ghana

**Reviewer** **#3**

**First question**

• The manuscript indicates that GeneXpert was the primary method used to validate DR-TB. However, the text does not specify what proportion of the study population underwent this testing. What percentage of the total population was tested for susceptibility to anti-TB drugs?

**Follow-up question**

**Reviewer** **#3: My questions have not been addressed fundamentally.** Most important of them is that the primary objective of the study is to assess the prevalence of drug-resistant tuberculosis (DR-TB) in Ghana, with a conclusion stating that the prevalence is low.
However, the authors did not conduct their own investigation of the prevalence of DR-TB. In their response, they claim that this was not the aim of the study, but this contradicts the text of the manuscript and its conclusion. The authors only refer to prevalence data reported by other sources, which raises concerns about how the data was collected. In a scientific study, it is essential to provide such details clearly.

**Response**

**Data Sources and Methodology**

The study utilized secondary data obtained from the District Health Information Management System 2 (DHIMS2) and existing records from DR-TB treatment registers at selected treatment centers. The prevalence of drug-resistant tuberculosis (DR-TB) was calculated based on TB case data recorded in DHIMS2 and the treatment registers within the study areas.

Importantly, the study did not involve active field screening of clients. Instead, it relied on data already captured through routine health service delivery. DHIMS2 serves as Ghana’s central repository for health data, aggregating monthly indicators from hospitals, health centers, and community-based health planning and services (CHPS) across the country.

**TB Screening Policy Context**

According to the Ghana Health Service (GHS) policy on TB case detection, all health facilities are expected to screen at least 10% of outpatient department (OPD) attendance for TB. Screening may also be extended to community settings such as churches, mosques, and other public venues when feasible. However, actual screening coverage varies across facilities—some may exceed the 10% threshold, while others may fall short. Due to this variability, the study was unable to determine a reliable percentage of the total population screened in the selected areas. Consequently, this metric was not reported.

**Limitations**

The prevalence of DR-TB reported in this study reflects only the cases that were tested and documented in the study areas. The study did not conduct susceptibility testing on the broader population potentially exposed to anti-TB drugs.
